# Integrating Personality Research and Animal Contest Theory: Aggressiveness in the Green Swordtail *Xiphophorus helleri*

**DOI:** 10.1371/journal.pone.0028024

**Published:** 2011-11-30

**Authors:** Alastair J. Wilson, Marloes de Boer, Gareth Arnott, Andrew Grimmer

**Affiliations:** 1 Institute of Evolutionary Biology, University of Edinburgh, Edinburgh, United Kingdom; 2 Institute of Biodiversity, University of Glasgow, Glasgow, United Kingdom; University of Plymouth, United Kingdom

## Abstract

Aggression occurs when individuals compete over limiting resources. While theoretical studies have long placed a strong emphasis on context-specificity of aggression, there is increasing recognition that consistent behavioural differences exist among individuals, and that aggressiveness may be an important component of individual personality. Though empirical studies tend to focus on one aspect or the other, we suggest there is merit in modelling both within- and among-individual variation in agonistic behaviour simultaneously. Here, we demonstrate how this can be achieved using multivariate linear mixed effect models. Using data from repeated mirror trials and dyadic interactions of male green swordtails, *Xiphophorus helleri*, we show repeatable components of (co)variation in a suite of agonistic behaviour that is broadly consistent with a major axis of variation in aggressiveness. We also show that observed focal behaviour is dependent on opponent effects, which can themselves be repeatable but were more generally found to be context specific. In particular, our models show that within-individual variation in agonistic behaviour is explained, at least in part, by the relative size of a live opponent as predicted by contest theory. Finally, we suggest several additional applications of the multivariate models demonstrated here. These include testing the recently queried functional equivalence of alternative experimental approaches, (e.g., mirror trials, dyadic interaction tests) for assaying individual aggressiveness.

## Introduction

Aggression is widespread in animals and occurs most commonly among conspecifics in relation to competition for resources such as food, territory or mating opportunities [1]. Given its role in mediating competitive interactions it is unsurprising that average levels of aggression often vary within populations, for example increasing with competitor density [2] or as a resource becomes limiting [3]. Moreover, aggressive behaviours expressed by individual are typically plastic. Motivated in particular by game theoretic models, there has been enormous interest in the circumstances under which individuals might choose to aggressively escalate a conflict [4], [5], and the information they might use to inform such choices [6], [7], [8]. However, despite this emphasis on context-specificity, recent empirical studies have also demonstrated consistent among-individual differences in aggressiveness in many taxa [9], [10], [11]. This finding is consistent with the view of aggression as one component of individual personality [12], [13]. Although context-specific behaviour and personality differences are in no sense mutually exclusive, most empirical studies to date have focused on only one or other of these phenomena. We suggest that a more complete understanding of the causes and consequences of aggression will be obtained by the use of analytical approaches that more readily accommodate both. Here we highlight how this can be achieved using linear mixed effect models, and provide an empirical demonstration using data from behavioural trials on the green swordtail *Xiphophorus helleri*.

Aggressiveness can be viewed as a latent characteristic that varies among individuals in a population (e.g., as one axis of overall personality [13]). However, defined in this way, individual aggressiveness is not directly observable but is usually inferred from observations of specific agonistic behaviours (e.g., threats, bites, displays) expressed towards one or more conspecifics in a social context. Importantly, we generally expect that the expression of these specific agonistic behaviours will be determined in part by features of the “opponent” towards which aggression is directed [14], [15]. In the simplest case the (perceived) presence of an opponent is normally required to elicit any agonistic behaviour. However, game theory also predicts that individuals should employ assessment strategies to determine their likelihood of winning a contest, or resource holding potential, RHP, (*sensu* [5]). Information gained can then be used to inform behavioural decision making [5], [16]. There is certainly widespread empirical evidence that the aggressive behaviour expressed by contesting individuals is modulated by differences in RHP including body size and development of weaponry [1]. However, the underlying assessment strategies may be complex and difficult to determine [17], [18]. For example, an individual may have access to information about its own state, termed “self-assessment”, or may also be able to gather information about the state of its opponent relative to its own ability, termed “mutual-assessment” (see [19] for review). Negative relationships between RHP difference and contest escalation (or duration) have been widely claimed as evidence for mutual assessment [20], [21], [22] although this pattern is actually consistent with self-assessment as well [23]. Moreover, while mutual assessment increases the likelihood of individuals avoiding costly defeats, it may be that assessing opponent state is costly, in terms of energy use, time and increased risk of predation. As such, self-assessment may be an efficient strategy to settle contests in some situations, with the importance of this strategy being increasingly recognised [19].

The objective of this paper is to empirically demonstrate an analytical framework that allows proper integration of personality studies with tests of existing contest theory predictions. Our rationale is that observed variation in contest behaviour likely arises from both personality variation and from plastic, or “context specific”, effects. Note that we use the term “context specific effect” in a very general sense to refer to sources of within-individual behavioural variation (e.g, opponent RHP, motivational state of an animal, experimental protocol applied). Rather than attempting to isolate these components, a more complete description of agonistic behaviours may result from application of an analytical framework capable of modelling both simultaneously. This integration can be achieved through the use of linear mixed effect models [24], which, though not particularly novel in behavioural research generally, have received surprisingly limited application in studies on aggression to date. In a simple case, with repeated measures on focal individuals, inclusion of focal identity as a random effect allows trait variance to be decomposed into within- and among-individual components (permitting estimation of repeatability), while also allowing the influence of covariates of known or hypothesised importance (e.g., self and/or opponent RHP) to be tested. However, such models can be usefully extended by empiricists interested in aggression in at least two ways. Firstly, since focal behaviour is expected to be influenced by opponent phenotype in a dyadic contest, we estimate “opponent repeatability” which can be considered as the tendency of specific opponent individuals to elicit consistent behavioural responses across focal individuals [15]. Secondly, sharing the view expressed by others that a full understanding of personality requires knowledge of the relationships among specific behaviours used to assay it and the stability of these relationships across contexts [13], [25] we extend our mixed effect modelling to a multivariate framework.

Multivariate models allow estimation and testing of correlation structures that are hypothesised to exist among behavioural traits. Just as variance can be decomposed into within- and between individual components for a single trait, covariance among traits can be similarly partitioned. Here we take this approach to explore the extent to which among-individual variation in a suite of specific agonistic behaviours can be viewed as arising from a single axis of variation in latent aggression. We also test the (within-individual) relationships among behavioural traits expressed under two experimental settings commonly used for studies of aggression in fish, dyadic trials against a live opponent and mirror tests. This allows us to test whether individual aggression as inferred from mirror trials is a useful predictor of aggression when confronted by a live opponent, a question that has recently been raised in the literature [26].

The green swordtail, *Xiphophorus helleri*, provides a well-known fish model for studies of dominance and aggression (see [27] and references therein), with males competing in contests over mates and/or food resources [28]. These contests can incorporate both ritualized displays and direct fighting [29], [30]. Extensive work in this (and closely related) species, has shown that males evaluate opponents in several ways including: assessment of vertical pigment ‘bars’ on their flank [31], “social eavesdropping” (observing non-self contests) [32] and visual assessment of sword length and body size [33]. Prior work on *Xiphophorus* therefore provides an expectation that agonistic behaviours expressed by individual males will depend, at least in part on “opponent effects” as well as on any among-(focal) individual variation in the latent character of aggression. Here we illustrate the application of univariate and multivariate mixed models to data from a captive population of swordtails by testing three specific hypotheses: 1) that there are repeatable among-individual differences in agonistic behaviour displayed by male swordtails across contexts (i.e. different opponents and/or experimental designs), 2) that different traits used as indicators of aggression are positively correlated at the within-individual level (both within and across-experimental designs) consistent with an important axis of among-individual variation in aggression, and 3) that observed behaviour directed towards a live conspecific is best explained not as a manifestation of focal aggression alone, but is also dependent on assessment strategies as predicted by contest theory.

## Materials and Methods

### Ethics statement

All work was approved by the University of Edinburgh local ethical review committee and carried out under license granted by the Home Office (UK) under the Animals (Scientific Procedures) Act 1986. Criteria were in place to terminate any trial immediately in the event of physical injury or other overt signs of distress (e.g., greatly raised opercular beat, failure to escape aggression from an opponent, abnormal swimming behaviour). No animal received injury as a result of the experiments and it was not necessary to terminate any individual trial. At the end of data collection all fish were released from the Animals (Scientific Procedures) Act 1986 (UK) following veterinary inspection and were re-homed as pets.

### Fish husbandry

Thirty commercially bred mature male swordtails were sourced from a tropical fish retailer. Nominally designated as green swordtails, *Xiphophorus helleri*, fish obtained were of four colour strains designated as “marigold”, “green”, “wagtail” and “red”, in order to facilitate individual identification during behavioural trials. These domestic fish have an unknown history of artificial selection, (probable) hybridisation with congenerics, and adaptation to captivity. In very broad terms we expect patterns of behavioural (co)variation to be similar to those that might be expressed by wild-counterparts subject to identical trials. Although we do expect that patterns of behavioural (co)variation will be broadly similar, we caution that results from this study should not be viewed as directly informative for the behavioural ecology of wild fish.

Each fish was assigned at random to one half of a partitioned home tank (one of 15 tanks measuring 40×40×30 cm and divided into two equal volumes using a transparent glass partition). Individuals were thus physically (but not visually or chemically) isolated from each other. Home tanks were enriched with rocks and plants, water temperature was maintained at 24°C, and a 12∶12 light∶dark cycle was imposed (lighting hours 0700-1900). Fish were fed on commercial flake food twice daily, supplemented with occasional feeding of live daphnia and frozen brine shrimp. Prior to the end of mirror trials, space constraints necessitated rehousing the fish in groups of 3–4. This change in housing regime is controlled for statistically in our analyses (see below).

### Experimental design and data collection

Behavioural and morphological data were collected between 9^th^ February and 1^st^ April 2010. Fish were subject to repeated trials of two different experimental protocols: a mirror test to assay aggression, and a dyadic interaction test against a live opponent (details below). Dyadic trials were completed prior to beginning mirror trials. Based on simulation-based power analyses (Appendix S1), four trials of each type per focal fish were planned, this being sufficient to detect repeatabilities as low as 0.2 with an estimated power of 0.70 (Figure S1), rising to an estimated power of 0.99 for repeatabilities above 0.35 (approximately the average repeatability reported for behavioural traits [34]). The final data structure collected deviated slightly from this due to some mortality (one fish died prior to data collection, and one during). Thus behavioural data were collected for 29 individuals, with a mean of 3.6 mirror trials and 7.2 dyadic trials per fish. For the dyadic trials 16 fish were used eight times, 7 used seven times, 3 used six times and one fish observed on a single occasion. Analyses used are robust to unbalanced data sets and among-individual variation in trial number is not expected to affect results or conclusions (particularly since trial number had no significant effects on behavioural means; discussed below).

Behavioural tests were conducted in glass experimental tanks as described below, with water at 24°C. Tanks were visually screened from the experimenter and filmed from above using a Sunkwang C160 video camera mounted with a 5–50 mm manual focus lens. The water was replaced between subjects to prevent any influence of pre-existing chemical cues. No fish was subject to a repeated trial within 48 hours of its last use, a period which is validated to minimize effects of prior social experience and stress in swordtails [30]. After completion of all trials, behaviours were scored from video using the key-logger software Jwatcher 0.9. Specific behavioural traits were scored for the two different protocols according to an ethogram developed from previously published behavioural descriptions for this genus [35], [36]. A brief description of the traits is given below while a more detailed ethogram is presented in the supplemental materials (see Appendix S2).

#### Mirror test

**Figure 1:**
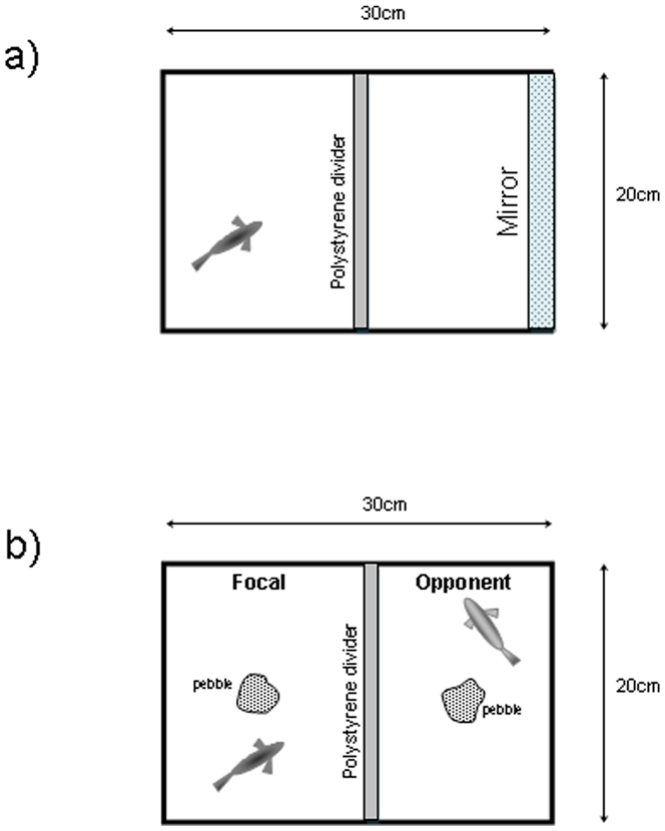
Above view of experimental tank set ups for start of (a) mirror tests and (b) dyadic interaction tests. Polystyrene dividers were removed at the start of each trial.

A single fish was removed from its home tank and placed in the left hand side of an experimental tank (filled to 8 cm), partitioned into two equal volumes with an opaque polystyrene divider. The fish was therefore visually isolated from a mirror placed at the right hand end of the tank (Figure 1a). After an acclimation period of 300 seconds, the divider was removed and a 180 second period recorded for analysis. The individual was then returned to its home tank. Each individual was tested twice during the period of isolated housing, and twice during the group housing period. Behaviours recorded as putative indicators of aggression were: (1) the latency to first approach of the mirror; (2) time spent in close proximity (≤5 cm) to the mirror; (3) time spent in lateral display to the mirror; and (4) the number of attacks made on the mirror. Approaches, attacks and display behaviour were defined operationally according to criteria presented in the ethogram (Appendix S2).

#### Dyadic interaction test

Pairs of fish were drawn at random with one individual being randomly designated as the *focal* individual, and another as the *opponent*. Pairs were accepted provided the following conditions were met: 1) no dyad was used more than once (irrespective of focal and opponent designations within the pair); 2) no dyads involving the two fish from a single partitioned home tank were allowed; and 3) no individual was in more than 8 dyads, with a maximum of 4 repeats as the focal individual. For each trial the two fish were transferred to an experimental tank partitioned into two halves by an opaque polystyrene divider and filled to a depth of 8 cm. The designated *focal* was placed in the left hand partition and the *opponent* in the right. Pebbles of (approximately) equal size and relative position were present in each side to provide spatial orientation and cover (Figure 1b).

After a 300 second acclimation period the divider was removed and the subsequent 445 seconds recorded for video analysis. Both fish were then returned to their home tanks. We used colour morph and pattern variation, coupled with visible differences in gross morphology (e.g., size, caudal sword length) to discriminate between *focal* and *opponent* individuals during the trial. Focal behaviours recorded as putative indicators of aggression were: (1) number of approaches to the opponent; (2) number of attacks on the opponent; (3) number of tail beats, (4) time spent in lateral display to the opponent, (5) latency to first aggressive action (i.e., any of the above mentioned behaviours); and (6) latency to attack (recorded as 445 seconds if no attack was observed). In addition we recorded two behaviours putatively indicative of defence (or avoidance) rather than aggression. These were the number of retreats (a slow controlled movement away from the opponent) and the number of flees (a much more rapid movement away from the opponent which was only observed following an attack by the latter). Behaviours were defined operationally according to criteria presented in the ethogram (Appendix S2) and were, as far as possible, defined so as to be analogous to those recorded in the mirror trials. However, tail beat display was recorded in dyadic interactions only as it was not observed during mirror trials (possibly as a consequence of shorter observation periods). Note also that since the observation periods differ between the protocols (180 seconds for mirror trials and 445 seconds for the dyadic) direct comparison of trait means is not appropriate (i.e., we expect, all else being equal, more aggressive actions in a longer observation period regardless of the stimulus type). However, here our hypotheses concern dimensionless parameters (specifically repeatabilities and correlations) with no necessary dependence on observation period (but see later discussion related to this point).

#### Morphological traits

Morphological data used for analyses were collected on a single sampling occasion at the end of the behavioural testing period. All fish were anaesthetised using a solution of MS222 100%w/w (buffered to neutral pH with sodium bicarbonate), weighed by electronic balance (±0.01 g) and photographed. Standard length (the distance from the anterior end to the last scale on the caudal peduncle along the midline) and caudal sword length (defined here as the distance along the dorsal edge of the sword from distal tip to the main lobe of the caudal fin) were measured from digital images using the morphometric software tpsDig. To avoid subjecting fish to repeated anaesthesia, we assumed morphology to be constant within-individuals across the study period. Consistent with this assumption, data collected by placing non-anesthetised fish in a beaker of water placed on the electronic balance (up to 8 weights per individual) provided no evidence of weight change during the course of the study (data not shown).

### Analysis and statistical modelling

All data were analysed using linear mixed effect models solved by restricted maximum likelihood and implemented in ASReml (Version 3). For the interested reader a didactic presentation of the general approach taken, together ASReml code for fitting the models is available at http://www.wildanimalmodels.org/tiki-index.php?page=Modelsforsocialdominanceandcompetition. In order to better meet the assumption of residual normality all behavioural traits were square root transformed prior to analysis. In addition, to ease interpretation of the multivariate analysis we multiplied the square roots of latency to aggression and latency to attack by −1 such that for all traits higher values correspond to greater aggression.

#### Estimation of behavioural repeatabilities and opponent identity effects

To test the hypothesis that behavioural traits associated with aggression are repeatable within individuals we fitted univariate models of each behavioural trait with the trait mean as a fixed effect and focal identity as a random effect. For traits assayed in the mirror tests we also included a fixed effect of housing regime (as a two level factor) in addition to the overall trait mean (μ), in order to account for any change caused by the switch from isolated to group housing such that trait y expressed by individual *i* in trial *k* was modelled as:
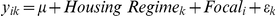



The repeatable individual effects *Focal_i_* are not directly observed, but are assumed to be normally distributed with a variance to be estimated as V_F_, the among-(focal) individual variance component which is estimated by fitting the mixed model. We make the standard assumptions that residual errors (*ε_k_*) are normally distributed and uncorrelated across observations, and estimate the residual variance V_R_ (which corresponds to the within-individual component of trait variance). We then estimate the focal repeatability R_F_ as the proportion of total phenotypic variance (V_P_) explained by individual identity such that R_F_ = V_F_/V_P_ (and V_P_ is simply estimated as the sum of V_F_ and V_R_).

For traits assayed in the dyadic interaction tests we modelled the observation from test k on individual *i* with opponent *j* as:
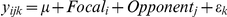
Here housing regime was not included since all fish were kept in isolation for the dyadic interaction testing period, but an additional random effect of opponent identity was included. Opponent identity effects were assumed to be normally distributed with mean zero and variance V_O_ (the among-opponent individual variance) to be estimated. We then estimated focal and opponent repeatabilities (R_F_ and R_O_) respectively, as the ratio of the corresponding variance component to total V_P_ (now estimated as the sum of V_F_, V_O_ and V_R_). R_O_ can be interpreted as the proportion of variance in a behavioural trait expressed by the focal individual that is attributable to the identity of its opponent [15].

Note that our hypotheses are entirely agnostic to the presence of carryover effects [37] that may arise from trial order (e.g. habituation), or experience (e.g., winner and loser effects; [38]). All individuals experienced trials of each experimental type in the same order such that this will not be a source of among-individual variance. Preliminary analyses also showed no significant effect of trial number on traits recorded in dyadic tests. After conditioning on housing regime (which is confounded with order) this was also true for the mirror trial data. Thus there is no evidence of habituation at the population level. However, it is certainly true that focal individuals encounter different sequences of opponents during the dyadic trials, and that the experience from one encounter may influence behaviour in a subsequent contest. Winner and loser effects have been well documented in swordtails, although they are not generally expected to persist beyond 48 hours [30] which was the minimum interval between repeated trials here. If present, these or other forms of carryover effect are not expected to bias our statistical hypotheses but do have implications for biological interpretation (see discussion).

#### Estimation of among-trait correlation structure

We then fitted multivariate models to estimate the among-trait correlation structure of repeatable individual effects. Usefully this allows both the estimation of among-trait correlations for focal effects (r_F_) and opponent effects (r_O_). Furthermore, where focal and opponent effects are present, it is also possible to estimate the correlation between them, both within and between-traits. Thus one can test the within-individual correlation between, for instance, a focal individual's tendency to attack an opponent and an opponent's tendency to induce fleeing in a focal animal.

We limited our multivariate analysis to that subset of behavioural traits for which V_F_ and/or V_O_ was significantly greater than zero (at α = 0.05) based on univariate analyses. We then modelled the full variance-covariance matrix, subsequently denoted **I**, among all individual-level (focal and opponent) random effects that were supported by the univariate models described above. **I** was then rescaled to the corresponding correlation matrix **I_r_**. To better facilitate model convergence and allow standard errors for all correlations to be quantified, **I** was estimated without any constraints on parameter space (i.e. estimates of at r>|1| are possible in **I_r_**).

To assess whether the overall pattern of multivariate behavioural variation among individuals is qualitatively consistent with a strong axis of variation for aggression, we first adjusted our mixed model estimate of **I_r_** to the nearest positive definite matrix (i.e. all r≤|1|) as determined by the R function *nearPD* algorithm [39]. We then performed a principal components analysis (PCA) by Eigen decomposition. Since **I_r_** is estimated with uncertainty, statistical inference on its principal components is non-trivial (though not impossible; see [40] for related discussion) and we therefore use the PCA only to provide a descriptive summary of the correlation structure among the repeatable components of the traits. Finally, we expanded the multivariate model to also include the morphological traits as response variables, allowing estimation of the correlations between size (and ornament size) and repeatable components of individual behaviour. This estimates potential relationships between behaviour and morphology without assuming any directional causality (see discussion).

#### Context specific opponent effects: the influence of RHP

We fitted two additional sets of univariate linear mixed effect models to test whether focal aggression varies as a function of focal and/or opponent resource holding potential (RHP). Firstly we tested the prediction that focal behaviour should vary with asymmetry in RHP estimated as relative body size. Specifically we predict focal individuals will tend to exhibit more “escalated” behaviour when they are the larger member of the dyad, for example by spending more time displaying and/or by attacking an opponent more frequently and more rapidly. Weight and standard length were highly correlated (see results) and we chose to use relative weight, calculated as ln(*WT_i_/WT_j_*) to measure size asymmetry in each trial. We then assigned trials to one of three classes based on the tertiles of this metric: 1 – focal *i* is smaller than opponent *j* (n = 35); 2 - focal and opponent of (approximately) similar size (n = 35); 3 - opponent is larger than focal (n = 34). The distributions of (untransformed) focal behaviour were visually compared across relative weight classes using box plots of the raw data, and statistically compared by refitting the univariate mixed effect models of transformed data after adding relative weight class (*relWT*) as a fixed effect (3 levels) such that:
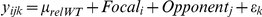



Secondly, we modelled the influence of RHP by including the partial regressions on absolute focal and opponent weight rather than the categorical relative weight class. Relying on a composite measure of RHP asymmetry has been shown to produce spurious results in terms of examining the RHP assessment strategy used [23]. To overcome this problem and correctly discriminate between mutual and self assessment, it is necessary to examine the influence of individual contestants RHP [18]. Studies typically model the effects of winner and loser RHP on measures of contest cost (duration or intensity). However, recently [41] modelled the effects of focal and opponent RHP on aggressiveness to examine visual opponent assessment in convict cichlid fish, and this is the approach used here.
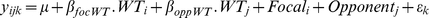



For both sets of models we estimated the corresponding “adjusted repeatabilities” (*sensu* [42]) for each response trait. The adjusted repeatability can be interpreted as the proportion of variance not explained by the fixed effects that is attributable to individual identity [42], [43].

#### Statistical testing

In all models we assessed the significance of fixed effects using Wald F-tests implemented in ASReml. For univariate models we tested the significance of V_F_ and (where fitted) V_O_ using one-tailed likelihood ratio tests, in which the log-likelihood of a model with focal identity (or opponent identity) included as a random effect was compared to the corresponding reduced model. The test statistic, calculated as twice the difference in model log-likelihoods is assumed to have a distribution corresponding to a 50∶50 mix of chi squared distributions having 0 and 1 DF respectively [44]. To test the within-individual correlations estimated under the multivariate models, we used a series of bivariate models for convenience. Specifically we compare the likelihood of a bivariate model in which the within-individual correlation was freely estimated to one in which it was constrained to equal zero. A two-tailed test was then performed, with the test statistic (again calculated as twice the difference in model log-likelihoods) assumed to be distributed as *Χ* ^2^_1DF_. We present P-values with no correction for multiple testing while acknowledging that the number of traits (and corresponding tests) raises concerns of Type I error. However traditional solutions (e.g., Bonferroni) introduce further difficulties [45] and are inappropriate given the strong *a priori* expectation of non-independence among test statistics (i.e. traits were chosen precisely because they are hypothesised to reflect a single axis of latent variation in aggressiveness). Although not implemented here, one potentially useful strategy would be to formulate “global” statistical hypotheses about the structure of the **I** (or **I_r_**) matrix as opposed to its individual elements.

## Results

### Focal and opponent repeatabilities

Univariate models confirmed our hypothesis that individuals show repeatable differences in behavioural traits chosen as putative indicators of underlying aggression. Across all traits tested estimates of focal repeatabilities R_F_ ranged from 5.4% to 42.5%, with the among-individual component of variance (V_F_) being significantly greater than zero (at α = 0.05) for 9 of the 12 traits tested (Table 1). In the mirror trials we found no statistical support for among-focal variance in latency to approach the mirror, while in the dyadic trials neither the number of approaches made nor the number of flees were significantly repeatable. In contrast, we did not generally find statistical support for the presence of repeatable opponent identity effects on traits observed in dyadic trials (Table 1). R_O_ estimates were generally low (ranging from zero to 17.4% with a median of 7%) and, with a single exception, V_O_ was not significantly greater than zero. The number of flees performed by a focal individual was the only trait for which non-zero opponent repeatability was statistically supported (R_O_ = 0.174 (0.102), P = 0.031). Interestingly, this means that while the focal identity is not a significant predictor of its tendency to flee in dyadic trials, the identity of the opponent is.

**Table 1:** Estimated variance components (with standard errors in parentheses) for behavioural traits.

Trial type	Response	V_F_	P	R_F_	V_O_	P	R_O_	V_R_
Mirror	−√(latency to approach mirror)	0.979 (0.860)	0.099	0.123 (0.103)				6.96 (1.14)
	√(time at mirror)	2.22 (1.34)	0.017	0.200 (0.107)				8.87 (1.45)
	√(time displaying at mirror)	3.04 (1.15)	<0.001	0.425 (0.106)				4.11 (0.67)
	√(no. attacks on mirror)	0.346 (0.229)	0.029	0.172 (0.104)				1.66 (0.271)
Dyadic	√(no. approaches)	0.120 (0.136)	0.167	0.083 (0.091)	0.171 (0.147)	0.091	0.119 (0.097)	1.15 (0.221)
	√(no. attacks)	0.560 (0.255)	0.001	0.292 (0.106)	0.030 (0.139)	0.412	0.016 (0.073)	1.33 (0.252)
	√(no. tail beats)	0.185 (0.133)	0.043	0.148 (0.100)	0.000 (−)^1^	0.500	0.000 (−)^1^	1.06 (0.170)
	√(time displaying)	1.82 (1.01)	0.008	0.208 (0.102)	0.705 (0.775)	0.153	0.081 (0.086)	6.22 (1.18)
	−√(latency to aggression)	2.21 (1.56)	0.042	0.157 (0.103)	0.000 (−)^1^	0.500	0.000 (−)^1^	11.8 (1.91)
	−√(latency to attack)	12.6 (5.47)	<0.001	0.316 (0.106)	3.00(3.20)	0.145	0.075 (0.079)	24.4 (4.70)
	√(no. retreats)	0.202 (0.121)	0.015	0.191 (0.103)	0.067 (0.092)	0.212	0.063 (0.086)	0.789 (0.148)
	√(no. flees)	0.061 (0.102)	0.270	0.054 (0.088)	0.197 (0.128)	0.031	0.174 (0.102)	0.878 (0.170)

The among-focal variance (V_F_), and residual variance (V_R_) are shown for all traits along with the among-opponent variance (V_O_) for traits observed in dyadic trials. Also shown are focal and opponent repeatabilities. P values denote the statistical significance of V_F_ and V_O_ respectively and are derived from 1-tailed likelihood ratio tests (see text for details).

1 With models constrained to yield permissible (i.e. non-negative) variance estimates this parameter was bound at the edge of parameter space. Under these conditions standard errors are non-estimable.

### Among-trait correlation structure of repeatable effects

Multivariate modelling also provided evidence of significant correlations among the individual level (repeatable) effects on different traits. Among behavioural traits assumed *a priori* as putative indicators of aggression, correlations were generally positive where significant (Table 2). For instance, within the dyadic trials, estimates of r_F_ indicate that those individuals that attack opponents more often also tend to perform more tail beats, spend more time in lateral display, and tend to have a shorter latency to attack. Furthermore, significant positive correlations were detected between focal effects on aggression traits and opponent effects on fleeing (Table 2). Since we did not observe an individual fish to flee except as a response to being attacked, it is expected that a fish that consistently causes another to flee will be one that consistently carries out more attacks. This expectation is supported by the estimated within-individual correlation (r_FO_) of 1.13 (0.397) between the repeatable focal tendency to attack and the repeatable opponent tendency to induce fleeing.

**Table 2:** Within-individual correlation structure among morphological and behavioural traits.

	Weight	Standard length	Sword length	√(time at mirror)	√(time displaying at mirror)	√(no. attacks on mirror)	√(no. attacks)	√(no. tail beats)	√(time displaying)	−√(latency to aggression)	−√(latency to attack)	√(no. retreats)
**Standard length**	0.947***											
	(0.020)											
**Sword length**	0.359^+^	0.368*										
	(0.168)	(0.166)										
**√(time at mirror)**	0.015	−0.068	−0.429^+^									
	(0.284)	(0.282)	(0.258)									
**√(time displaying at mirror)**	0.238	0.209	−0.123	0.761**								
	(0.216)	(0.217)	(0.223)	(0.220)								
**√(no. attacks on mirror)**	−0.746**	−0.824**	−0.155	−0.182	0.129							
	(0.257)	(0.247)	(0.303)	(0.551)	(0.361)							
**√(no. attacks)**	0.428^+^	0.298	−0.094	0.126	0.686*	−0.037						
	(0.214)	(0.231)	(0.245)	(0.359)	(0.217)	(0.393)						
**√(no. tail beats)**	0.244	−0.017	−0.301	−0.038	0.389	−0.385	0.786*					
	(0.298)	(0.305)	(0.292)	(0.449)	(0.349)	(0.482)	(0.211)					
**√(time displaying)**	0.609*	0.388	−0.036	0.235	0.102	−0.216	0.759*	0.258				
	(0.232)	(0.261)	(0.278)	(0.408)	(0.338)	(0.449)	(0.263)	(0.417)				
**−√(latency to aggression)**	−0.210	−0.365	−0.116	0.072	−0.018	0.753^+^	0.150	−0.291	0.152			
	(0.290)	(0.278)	(0.294)	(0.431)	(0.355)	(0.432)	(0.395)	(0.508)	(0.436)			
**−√(latency to attack)**	0.562*	0.432^+^	0.195	0.107	0.518^+^	−0.189	1.01***	0.728*	0.695*	0.183		
	(0.190)	(0.212)	(0.237)	(0.358)	(0.251)	(0.387)	(0.073)	(0.289)	(0.260)	(0.380)		
**√(no. retreats)**	−0.287	−0.467^+^	−0.195	0.694^+^	0.342	1.25**	0.381	0.398	−0.187	0.523	0.086	
	(0.280)	(0.264)	(0.283)	(0.374)	(0.329)	(0.423)	(0.378)	(0.478)	(0.438)	(0.433)	(0.382)	
**√(no. flees)** 1	0.645*	0.620*	0.206	0.315	0.743*	0.181	1.13**	0.513	1.21**	−0.184	1.20**	−0.493
	(0.268)	(0.264)	(0.292)	(0.426)	(0.295)	(0.463)	(0.397)	(0.475)	(0.413)	(0.447)	(0.491)	(0.452)

Bold font denotes correlation estimates significantly different from 0 at P≤0.05, while * and ** denote estimates significant at P≤0.01 and P≤0.001 respectively. Marginally non-significant correlations are also indicated (^+^ denotes 0.10≤P<0.05). Standard errors are indicated in parentheses.

1 Opponent effect is interpretable as the (transformed) tendency to cause other fish to flee.

Principal component analysis (eigen decomposition) of the correlation matrix revealed a first principal component that explained 45% of the variance in **I_r_** and had moderate loadings of a consistent sign on most of the agonistic behaviours (Table 3). Some exceptions to this pattern are evident however. For instance, latency to aggression (dyadic tests) and number of attacks on the mirror (mirror test) both had loadings close to zero on PC1. A similar result was obtained for number of retreats as observed in dyadic tests (for which we might naively have expected a loading coefficient of opposite sign to those of the agonistic traits). Interestingly, these three traits that were not captured in PC1 did however have high loadings of consistent sign on PC2 which accounted for a further 26% of the variance (Table 3).

**Table 3:** Loading coefficients for the first two principal components of the within-individual correlation matrix among repeatable behaviours.

Trial type	Response	PC1	PC2
Mirror	√(time at mirror)	−0.169	0.237
	√(time displaying at mirror)	−0.329	0.186
	√(no. attacks on mirror)	0.030	0.561
Dyadic	√(no. attacks)	−0.461	0.047
	√(no. tail beats)	−0.320	−0.080
	√(time displaying)	−0.369	−0.133
	−√(latency to aggression)	−0.012	0.466
	−√(latency to attack)	−0.456	−0.048
	√(no. retreats)	−0.058	0.580
	√(no. flees)^1^	−0.448	−0.110

PC1 and PC2 account for 45% and 26% of the variance respectively.

1 Opponent effect is interpretable as the (transformed) tendency to cause other fish to flee.

As expected, positive correlations were found among the three morphological traits weight, standard length and sword length (with a particularly strong association between the two measures of body size, r = 0.947 (0.020)). There was little support for association between sword length and the repeatable components of behaviour, but in general large fish were more aggressive (Table 2). For example, large size as measured by either standard length or weight, was significantly (or marginally non-significantly) associated with more attacks, more time spent displaying, a shorter latency to attack, and a tendency to induce focal fleeing when acting as the opponent in dyadic trials (Table 2). Conversely, there was actually a negative correlation between an individual's size and his repeatable tendency to attack the mirror (estimates of r_F_ = −0.746 (0.257), P<0.001 or r_F_ = −0.824 (0.247), P<0.001 between weight or standard length respectively and √(no. attacks on mirror)).

### Context specific opponent effects: the influence of relative and absolute opponent size

**Figure 2:**
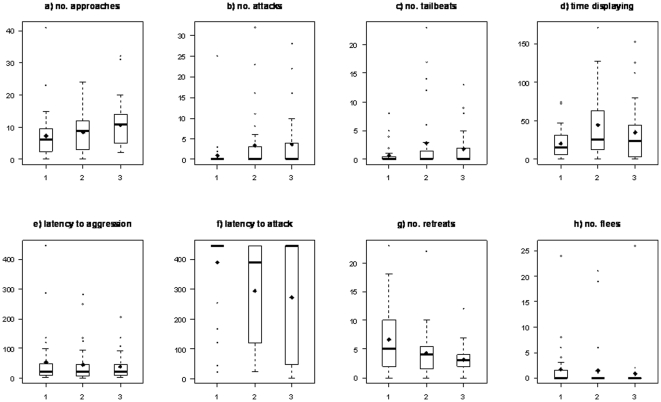
Box plots showing distribution of untransformed focal trait observations in the dyadic interaction trials by relative size class. Three classes of relative size class were assigned such that 1 = focal smaller that opponent, 2 = approximately similar size, 3 = focal larger than opponent (see text for details). Horizontal lines indicate the trait median, boxes contain the interquartile range, and whiskers extend to the most extreme data point which is no more than the interquartile range from the box. Outliers are indicated by circles and for comparison to the median the trait means are also indicated by black diamonds. (Note that due to non-independence of data across the relative size classes no statistical comparisons are made here).

Plotting the behavioural data distributions by relative weight class suggests that a number of the traits observed in dyadic trials are influenced by relative body size (Figure 2). For example, the average (median) number of approaches and attacks increases with focal size relative to the opponent (Fig. 2a,b), while the average latency to attack and the average number of retreats decrease (Figs. 2f, g). For both number of tail beats and time spent displaying, trait averages were highest for relative weight class two (i.e., when focal and opponent were of approximately similar size, Figs. 2c,d). There was no discernible pattern of change in latency to aggression or the number of flees across relative weight classes (Figs. 2e, h). In some cases statistical significance of these qualitative patterns was supported by the linear mixed effects models of transformed data. Thus, relative weight class was a statistically significant (at α = 0.05) predictor of (transformed) focal behaviour for √(no. attacks), √(time displaying), −√(latency to attack), and √(no. retreats) (Table 4). However the effect of relative weight classes was non-significant for √(no. approaches) and √(no. tail beats), although the latter was marginally non-significant (F_2_,_96.1_ = 2.72, P = 0.072). Adjusted (for relative weight class) focal repeatabilities were similar to the unadjusted values (comparison of estimates in Tables 1 and 4, median (R_F_ - R_F.adj_) = −0.0025, Wilcoxon signed rank test P = 0.726). Adjusted opponent repeatabilities were slightly lower on average than the unadjusted values although the difference was marginally non-significant (comparison of estimates in Tables 1 and 4, median (R_O_ – R_O.adj_) = 0.019, Wilcoxon signed rank test P = 0.059).

**Table 4:** Effect of relative weight class on focal behaviours in dyadic trials.

Response	Effect of relative weight category					Adjusted repeatabilities			
	Factor level	Predicted mean (SE)	F	DF	P	R_F.adj_	P	R_O.adj_	P
√(no. approaches)	1	2.41 (0.218)	2.39	2, 76.8	0.100	0.078 (0.094)	0.183	0.076 (0.095)	0.195
	2	2.67 (0.212)							
	3	3.07 (0.225)							
√(no. attacks)	1	0.332 (0.253)	3.99	2, 97	0.022	0.276 (0.107)	0.001	0.00 (−)	0.500
	2	1.11 (0.243)							
	3	1.13 (0.260)							
√(no. tail beats)	1	0.381 (0.201)	2.72	2, 96.1	0.072	0.152 (0.102)	0.041	0.00 (−)	0.500
	2	0.972 (0.195)							
	3	0.831 (0.206)							
√(time displaying)	1	4.09 (0.562)	4.04	2, 79.9	0.022	0.209 (0.108)	0.013	0.093 (0.093)	0.137
	2	5.94 (0.532)							
	3	4.90 (0.583)							
−√(latency to aggression)	1	−6.22 (0.698)	0.58	2, 96.4	0.560	0.184 (0.108)	0.027	0.00 (−)	0.500
	2	−5.32 (0.677)							
	3	−5.41 (0.716)							
−√(latency to attack)	1	−19.0 (1.18)	3.37	2, 73.5	0.040	0.268 (0.109)	0.002	0.040 (0.080)	0.300
	2	−15.8 (1.12)							
	3	−15.4 (1.22)							
√(no. retreats)	1	2.339 (0.19)	4.36	2, 74.7	0.017	0.218 (0.108)	0.009	0.041 (0.084)	0.306
	2	1.781 (0.182)							
	3	1.634 (0.196)							
√(no. flees)	1	0.693 (0.201)	1.33	2, 78.5	0.271	0.089 (0.098)	0.174	0.128 (0.102)	0.086
	2	0.441 (0.194)							
	3	0.237 (0.21)							

Three classes of relative size class were assigned such that 1 = focal smaller that opponent, 2 = approximately similar size, 3 = focal larger than opponent (see text for details). Also shown are adjusted focal and opponent repeatabilities (R_F.adj_ and R_O.adj_) with P-values from likelihood ratio tests of the corresponding variance components.

When linear effects of absolute weight (both focal and opponent), rather than relative weight class were modelled there was a general pattern of decreasing aggression with increasing opponent size (Table 5). The partial regression of (absolute) opponent weight on (transformed) focal behaviour was negative for five of the six traits used to assay focal aggression (significant in 3), and was significantly positive for both √(no. retreats) and √(no. flees). The partial regression of (absolute) focal weight on focal behaviour was significant for two traits, with heavier focal individuals spending more time displaying and having a reduced latency to attack (Table 5). On average they also attacked more frequently although this result was marginally non-significant (*β_focWT_* = 1.40 (0.715), P = 0.06). Adjusted (for absolute weight) focal repeatabilities were similar to unadjusted values (comparison of estimates in Tables 1 and 5, median (R_F_ - R_F.adj_) = −0.003, Wilcoxon signed rank test P = 0.945) as were adjusted opponent repeatabilities (comparison of estimates in Tables 1 and 4, median (R_O_ – R_O.adj_) = 0.008, Wilcoxon signed rank test P = 0.529).

**Table 5:** Effect of absolute focal and opponent weights on focal behaviours in dyadic trials.

Response	Effects of focal and opponent weight					Adjusted repeatabilities			
	Predictor	Beta (SE)	F	DF	P	R_F.adj_	P	R_O.adj_	P
√(no. approaches)	*β_focWT_*	0.471 (0.524)	0.8	1, 25.4	0.378	0.086 (0.095)	0.163	0.076 (0.095)	0.196
	*β_oppWT_*	−1.296 (0.52)	6.2	1, 25.7	0.019				
√(no. attacks)	*β_focWT_*	1.40 (0.715)	3.42	1, 26.3	0.06	0.272 (0.108)	0.001	0 (0)	0.500
	*β_oppWT_*	−1.23 (0.497)	6.15	1, 90.5	0.016				
√(no. tail beats)	*β_focWT_*	0.460 (0.542)	0.58	1, 26	0.404	0.158 (0.103)	0.037	0 (0)	0.500
	*β_oppWT_*	−0.724 (0.438)	2.73	1, 94.2	0.104				
√(time displaying)	*β_focWT_*	3.64 (1.32)	7.6	1, 24.6	0.011	0.126 (0.098)	0.073	0.146 (0.101)	0.050
	*β_oppWT_*	0.078 (1.36)	0.00	1, 25.1	0.955				
−√(latency to aggression)	*β_focWT_*	−1.28 (1.83)	0.49	1, 25.4	0.491	0.16 (0.105)	0.041	0 (0)	0.500
	*β_oppWT_*	−1.89 (1.47)	1.64	1, 93.9	0.206				
−√(latency to attack)	*β_focWT_*	8.80 (3.03)	7.87	1, 25.6	0.007	0.225 (0.106)	0.006	0.087 (0.089)	0.140
	*β_oppWT_*	−5.32 (2.55)	4.35	1, 24.7	0.047				
√(no. retreats)	*β_focWT_*	−0.610 (0.522)	1.12	1, 25.4	0.254	0.228 (0.109)	0.007	0.029 (0.08)	0.352
	*β_oppWT_*	0.915 (0.403)	5.16	1, 24.7	0.032				
√(no. flees)	*β_focWT_*	0.075 (0.475)	0.08	1, 24.3	0.875	0.105 (0.099)	0.126	0.114 (0.099)	0.104
	*β_oppWT_*	1.21 (0.482)	6.28	1, 25.4	0.019				

Partial regression coefficients of focal and opponent weight are denoted *β_focWT_* and *β_oppWT_* respectively. Also shown are adjusted focal and opponent repeatabilities (R_F.adj_ and R_O.adj_) with P-values from likelihood ratio tests of the corresponding variance components.

## Discussion

Our analyses confirmed that there are consistent, repeatable differences among male swordtails in agonistic behaviour. Broadly speaking, and with several exceptions and caveats (discussed below), our multivariate models also provide qualitative and quantitative support for the hypothesised major axis of variation in aggression among fish used for this study. Thus, not only were multiple agonistic behaviours repeatable, but we also found evidence of significant correlations among behavioural traits. This is consistent with the premise that there is an important axis of among-individual variation in latent aggression (and that at least some of the traits studied are useful assays of this axis). These results agree with a number of recent studies of aggression (and dominance) in *Xiphophorus* [46] and other fish taxa [47]. However, it is also possible that correlations among traits may occur because they form an escalatory sequence of behaviours. For example, in the sequential assessment model (SAM; [16]), activities are performed in a series of phases that reveal information about fighting ability. In this model contests begin with low-cost/low-intensity displays that are relatively unreliable indicators of RHP. As contestants become more closely matched the contest proceeds through a series of more escalated phases of activity that better indicate RHP. Thus a contest may proceed in a predictable fashion. There is empirical support for SAM in contests between male cichlid fish [21], whereby encounters had a consistent behavioural sequence, beginning with low intensity displays followed by bouts of tail beating, biting, mouth wrestling, and finally circling.

We also found support for the third of our hypotheses, namely that observed agonistic behaviours during dyadic contests should be dependent on assessment strategies predicted by contest theory [19]. While opponent repeatabilities were generally low and non-significant, there was evidence that focal fish are more likely to escalate agonistic behaviours (e.g. attack more) against an opponent smaller than themselves. When we simultaneously modelled effects of absolute focal and opponent weight (as per [41]) we found patterns that were consistent with a mutual assessment strategy but also a form of self assessment termed the cumulative assessment model (CAM; [48]) (for a review see [19]). That is, focal aggressiveness was positively related to own weight and also negatively related to opponent weight. We also found a positive relationship between focal body size and the repeatable component of several agonistic behaviours. Thus, in addition to showing an influence of opponent size (whether measured as absolute or relative opponent weight) on the observed behaviour within a trial, our data also show that bigger individuals are consistently more aggressive across trials. With mutual assessment contestants assess their opponent's RHP relative to their own, therefore requiring information about their own ability and that of an opponent. The CAM is a form of self assessment with contestants terminating the contest when accrued costs exceed an absolute individual threshold, and no direct information is gathered about the opponent [48]. However, unlike ‘pure’ self assessment (sensu [19]), in which costs accrue only as a result of each rival's own behaviour, in the CAM costs also accrue from the opponent's actions, and superior opponents are better at inflicting costs. Thus, in the CAM the decision to withdraw is influenced by both an individual's own RHP and the opponent's RHP. Consequently, the CAM produces the same relationship between contestant RHP and fight cost as predicted by mutual assessment. That is, the CAM will have the appearance of mutual gathering of information even though the decision is based on individual thresholds of cost. Distinguishing between assessment strategies is difficult [17], and requires further work involving staged interactions between RHP matched rivals (see [19]).

Thus, within a single modelling framework we have explored the among- and within-individual sources of variance in agonistic behaviour, and find evidence consistent with personality variation and with the predictions of contest theory. This highlights the important point that the presence of consistent behavioural differences among-individuals does not negate an important role for plasticity [49]. Of course the corollary is also true, most behavioural traits are considered to be highly plastic, but this does not mean they are not repeatable [34]. We focus the remainder of this discussion on issues arising from the mixed model approach advocated, using the current results as an example to highlight some of its strengths, limitations, and further applications.

### Repeatability, personality and interpretation of I_r_

Our analyses provide evidence for personality, defined for analytical purposes as a tendency for individuals to exhibit consistent differences in behaviour across multiple contexts [13]. This conclusion is based on the view that opponents of different identity (and RHP) represent different “contexts” under which agonistic behaviours are expressed. Repeatability, the statistical signature of this tendency, tells us that trait observations on the same individual are correlated but does not give any biological insight into why. Thus we cannot say if the personality variation detected arises from genetic effects, from differences in environments experienced (e.g., feeding regime, housing density in early life), or from variation in social experience prior to, or potentially during, this study. In the latter context we note that, the experience of winning (or losing) a contest, may itself increase the probability of that individual winning (or losing) a subsequent contest shortly after [38]. Thus, winner and loser effects will generate within-individual correlation (i.e. repeatability) of contest winning, and potentially of associated agonistic behaviours. Thus, we view carryover effects as a (potential) source of personality variation rather than as a confounding factor. This may be deemed undesirable for some research questions, in which case modifications to the repeatability models fitted here may be useful (e.g., inclusion of previous contest outcome as a fixed effect), and other types of mixed-model analyses could also be considered (e.g., autoregressive models [50]).

We have also argued that the multivariate correlation structure captured in **I_r_** is consistent with a major axis of variation in aggression, a latent character that may be thought of as one component of personality. However, this conclusion must be tempered by the fact that some correlation structure is an inevitable consequence of how traits are defined (e.g., time displaying to the mirror is necessarily a subset of time spent in proximity to the mirror). It is also important to note that positive correlations were not ubiquitous among putative assays of aggression and in some cases estimates of r_F_ were strikingly inconsistent with expectations. For example, an estimate of r_F_ = 1.248 (0.423) between the number of retreats (dyadic tests) and number of attacks on the mirror is counter to the intuitive expectation that a more aggressive individual would retreat less. Furthermore, PC1 accounted for slightly less than half of the total variance in **I_r_**, which is arguably smaller than we might expect given all traits were chosen with the express intent of assaying aggression.

It is therefore an oversimplification to say that all repeatable (multivariate) behavioural variation assayed here corresponds to a single axis of personality variation (i.e., aggression). Two non-exclusive possibilities follow from this. Firstly, we may conclude that attempting to reduce (co)variation in multiple agonistic behaviours to a single dimension is inappropriate. Secondly, we may question whether all of the putatively agonistic behaviours included in our analysis are equally relevant or valid as measures of aggression.

For instance, three traits, specifically latency to aggression, number of retreats, and number of attacks on the mirror do not load on the first principal component of **I_r_**, but rather on the second (which accounted for a further 26% of the variance). Limited data available from open field tests shows that time spent stationary is repeatable (R_F_ = 0.254 (0.109), P = 0.004) and correlated with an individual's inverse latency to aggression (r_F_ = −1.269 (0.419), P = 0.003), and tendency to attack the mirror (r_F_ = −0.729 (0.346), P = 0.074), though not with tendency to retreat. Thus, a *post hoc* interpretation might be that these traits are principally indicative of general activity level, a trait that has been used to investigate exploratory behaviour and/or boldness; [13], rather than aggression. Thus, our multivariate modelling strategy could (assuming the availability of pilot data) usefully be applied in the planning stages of a project to help decide which traits to focus on (e.g., a set of traits with high r_F_ if the aim is to study a single aspect of personality), or to mimimise redundancy of effort (e.g., avoid highly correlated traits if the aim is to study multiple axes of personality).

The structure of the correlation matrix **I_r_** is also informative for assessing the extent to which alternative experimental protocols reveal equivalent information about individual subjects. Assaying aggression by exposure to a stimulus designed to mimic a live opponent (e.g., a mirror, or “dummy” conspecific) has practical advantages, but the assumption that such stimuli elicit responses strictly comparable to those of a live opponent [51] has recently been questioned [52]. For example, Goulet and Beaugrand [53] reported that mirror test of *Xiphophorus* predicted victory but not the level of aggression in subsequent dyadic trials, while Arnott and Elwood [54] have argued that mirror trials in convict cichlids may be flawed because fish are unable to adopt the preferred mutual display orientation found in real contests [54]. In a different cichlid species, *Astatotilapia burtoni*, [26] found differences in immediate early gene (IEG) expression among localised areas of the brain when “fighting” a mirror as compared to a live opponent, and concluded that mirror responses may be more representative of fear than aggression.

Here we found that individuals that spend a lot of time displaying to the mirror also tend to attack a live opponent more often and more quickly, as well as inducing more flee responses. However, we also found that the within-individual correlation between time displaying at the mirror and time displaying to a live opponent is weak (r_F_ = 0.102 (0.338)), as is that between the tendency to attack a mirror and to attack a live opponent (r_F_ = −0.037 (0.393)). It was also notable that larger individuals attacked the mirror less, while attacking opponents more in the dyadic trials. This difference is consistent with a mutual assessment strategy (since a large focal is always confronted by an equally large “opponent” in a mirror trial, but more commonly by a smaller opponent in a dyadic test). This highlights the point that mirror trials can provide reliable information about an individuals's likely response to a live opponent, but careful choice of indicator traits may be critical and interpretation is not always straightforward. We also note that our interpretation with respect to stimulus type implicitly assumes that ranking of individuals is independent of observation period (since this also differed between mirror and dyadic interaction protocols). Censoring the dyadic data to include only the first 180 seconds (as used for the mirror trials) indicates this is not unreasonable. Specifically, for those traits that were repeatable (as indicated in Table 2), the within-focal individual correlations (SE in parentheses) between full and censored data sets are close to unity, ranging from 0.890 (0.286) for √(no. tail beats) to 0.999 (0.053) for √(no. attcks). In no case do the correlations differ significantly from +1 as would be expected if ranking was altered by use of the censored data (full results not shown).

A final question that arises for the interpretation of **I_r_** is the extent to which it captures variation in morphology as well as behaviour. Given the generally positive correlations between body size and repeatable effects on agonistic behaviours, our expectation is that PC1 of **I** will also map to size variation (i.e., more aggressive individuals being, on average, larger). We see no difficulty with this, particularly as the mechanistic basis of size-behaviour covariance is unknown. For instance, that large individuals tend to escalate conflicts can be explained as a consequence of the risks to themselves being (on average) less [19]. However, more aggressive individuals may dominate food resources during development and thus grow to a larger size, or a correlation between size and aggressiveness may reflect a shared dependence on an unmeasured trait (e.g., testosterone production). If appropriate to the hypotheses being tested, estimates of repeatability (and **I_r_**) can be readily conditioned on (or “adjusted” for) size. This is most readily done by inclusion of the size as a fixed covariate in the model, with the corresponding variance explained by size omitted from the estimate of V_P_. (i.e. as in our analyses of context specific opponent effects discussed below). However, it is important to recognise that this necessarily changes the biological interpretation of the analysis (see [42], [43] for related discussion).

### Repeatable and context-specific opponent effects

The estimates of R_O_ obtained here provide a measure of repeatable opponent influence on focal behaviour that integrates over the whole opponent phenotype. An advantage of this approach is that we can estimate the importance of opponent effects without prior knowledge of which specific traits influence focal behaviour [15]. However, if an opponent's influence depends on its relative, rather than absolute phenotype, it will be context specific and may not be detected through estimation of R_O_. With one exception, we found little statistical support for significantly repeatable effects of opponent identity, but there was wider support for context-specific effects of relative opponent size.

Significant R_O_ was detected for the focal response trait of fleeing. In fact, for this trait R_O_ was approximately three times the magnitude of R_F_. This finding is likely driven by a tight coupling between an attack by one individual and a flee by the other (not all attacks elicit flight behaviour, but fleeing is only observed in response to being attacked). Consistent with this view we found a strong positive correlation between an individual's repeatable tendency to attack and its tendency to induce fleeing. Although this treatment of correlated focal and opponent effects may be unfamiliar to some, it is really just a generalisation of a Bradley-Terry model [55] to traits other than binary contest outcomes. For contest winning, or dominance, if R_F_ is non-zero then it follows logically that we expect non-zero R_O_ (and a non-zero correlation between focal and opponent effects). This is because an effect that predisposes to (focal) winning when expressed in the focal individual, necessarily predisposes to (focal) losing when encountered in an opponent [56]. Thus, despite the general lack of support for R_O_ here, we suggest that modelling repeatable opponent effects on focal phenotype should have wide application. We also note that, regardless of statistical significance, including opponent identity as a random effect prevents pseudoreplication (since each opponent is used in multiple trials), and ensures the correct degrees of freedom are applied to tests of other model effects.

By comparison, our analyses provided wider support for context specific opponent effects of relative size. Where effects were statistically supported the predicted mean response was uniformly lowest (highest) for traits used to assay aggression (avoidance) when the focal individual was smaller (larger) than the opponent. Assuming size/weight to be a valid proxy for RHP, then escalated aggression is predicted when the contest is symmetric [57], [58], [59] and we found some qualitative support for this. For example the median latency to attack was actually lowest when both individuals were of similar size. Similarly, time spent displaying, and the number of tail beats performed were both higher in symmetric contests than when a focal was faced with a smaller opponent, a result that may be further suggestive of a mutual assessment strategy (i.e. individuals spend more time in activities useful for assessing relative RHP when a contest is symmetric). Previous authors have demonstrated that relying on a composite measure of relative RHP asymmetry is problematic, leading to spurious results in terms of discriminating between alternative assessment strategies [18], [23]. Therefore, we adopted the approach recommended by [41], of modelling the effects of each individual contestant's weight on focal aggressiveness. We found evidence that both focal and opponent RHP influence aggressive behaviour by the focal animal consistent with a mutual assessment or CAM strategy, and with previous work in swordtails [30], [32], [33].

### Summary

We have shown here that behavioural traits assayed through mirror tests and dyadic trials of male *Xiphophorus* are repeatable. We have also shown that repeatable, individual-level effects are correlated across traits both within- and across different experimental test types. This result is broadly consistent with the presence of an important axis of among individual variance in latent aggression. Furthermore, within the same analytical framework we also demonstrate context-dependent behavioural variation, and find evidence that focal individuals modify behavioural expression in response to the size of their opponent and relative to their own ability. Thus our modelling approach allows simultaneous testing of hypotheses relating to both the among- and within-individual components of (multivariate) behavioural variation. More generally, we highlight the utility of multivariate linear mixed effect models which, despite their limited application to date [37], offer powerful tools for the study of multivariate behavioural phenotypes.

## Supporting Information

Figure S1 **Estimated power curves for the detection of trait repeatabilities from a sample size of 30 individuals with 2 (dotted line), 3 (dashed line) or 4 (solid line) repeat observations per individual.** Power is estimated as the proportion of simulated data sets (with n = 200) for which the individual variance component is statistically significant at α = 0.05 based on a one-tailed likelihood ratio test (see main text for details). With a simulated repeatability of zero the power estimate is therefore an estimate of the type I error rate.
(DOC)

Appendix S1 **Simple power analyses for detection of trait repeatabilities.**
(DOC)

Appendix S2 **Ethogram of behaviours assayed in mirror tests and dyadic interaction tests.**
(DOC)
