# Supplementary material for: Integrating Personality Research and Animal Contest Theory: Aggressiveness in the Green Swordtail Xiphophorus helleri
Source: PLoS One. 2011 Nov 30;6(11):e28024. doi: 10.1371/journal.pone.0028024 (PMC3227624; doi:10.1371/journal.pone.0028024)
Supplement: Figure S1 — Estimated power curves for the detection of trait repeatabilities from a sample size of 30 individuals with 2 (dotted line), 3 (dashed line) or 4 (solid line) repeat observations per individual. Power is estimated as the proportion of simulated data sets (with n = 200) for which the individual variance component is statistically significant at α = 0.05 based on a one-tailed likelihood ratio test (see main text for details). With a simulated repeatability of zero the power estimate is therefore an estimate of the type I error rate. (DOC) [file pone.0028024.s001.doc]

**Supplemental figure 1:** Estimated power curves for the detection of trait repeatabilities from a sample size of 30 individuals with 2 (dotted line), 3 (dashed line) or 4 (solid line) repeat observations per individual. Power is estimated as the proportion of simulated data sets (with n=200) for which the individual variance component is statistically significant at α = 0.05 based on a one-tailed likelihood ratio test (see main text for details). With a simulated repeatability of zero the power estimate is therefore an estimate of the type I error rate.

Repeatability

Estimated power
