# Supplementary material for: Integrating Personality Research and Animal Contest Theory: Aggressiveness in the Green Swordtail Xiphophorus helleri
Source: PLoS One. 2011 Nov 30;6(11):e28024. doi: 10.1371/journal.pone.0028024 (PMC3227624; doi:10.1371/journal.pone.0028024)
Supplement: Appendix S1 — Simple power analyses for detection of trait repeatabilities. (DOC) [file pone.0028024.s002.doc]

**Supplemental Appendix 1**

**Simple power analyses for detection of trait repeatabilities**

Estimation of trait repeatabilities (and among-trait within individual correlations) requires that repeated measures on individuals are available. Although it is common for empiricists to estimate repeatabilities from just two measures per individual uncertainty around such estimates will often be large, particularly when the number of individuals is low. Since statistical power will increase (all else being equal) with the number of repeat measures on each individuals, we conducted a simulation-based power analysis to compare statistical power across a range of possible values for repeatability (0 to 1 in increments of 0.1) under scenarios of 2, 3, or 4 measures per individual.

**Approach**

For each combination of true repeatability and number of repeat measures, we simulated 200 sets of phenotypic observations. The phenotype of individual j observed on occasion k was simulated as:

*y_jk_ = I_j_ + ε_k_*

where *I_j_* is a repeatable effect, common to all observations on individual *j*, and is drawn from a normal distribution with mean of zero and specified variance (*σ^2^_I_*). Since we assume unit variance in *y*, *σ^2^_I_* is equal to the repeatability. *ε_k_* is a residual term drawn from a normal distribution with mean zero and variance (*σ^2^_R_*) equal to 1-repeatability.

For each simulated data set we then fitted a linear mixed effect model (using ASReml-R) containing individual as a random effect resulting in an estimate of the among-individual variance (V_I_). Statistical power was estimated as the proportion of the 200 simulated data sets for which V_I_ was deeded statistically significant at α = 0.05, based on a likelihood ratio test comparison to a reduced (null) model in which the random effect was not included. We conducted a 1-tailed test (i.e. H0: V_I_ = 0 against H_1_: V_I_ > 0), calculating the test statistic as twice the difference in model log-likelihoods and assuming this was distributed as a 50:50 mix of chisquared distributions with 0 and 1 degree respectively.

**Results**

Estimated power curves were saturated for true repeatabilities >0.8 regardless of the number of repeats per individual (Figure 1). However, for 30 individuals estimated power was low for plausible ranges of repeatability (e.g., 0.2-0.4) when only two repeat measures were made and this strategy was therefore rejected. Considerable gains in estimated power are expected by increasing the number of repeats to 3, and while the relative benefit of increasing from 3 to 4 was less we nevertheless opted to do this in order to maximise our probability of detecting repeatabilities of the order of 0.2 to 0.3. Given time constraints and an ethical requirement to minimise disturbance to the animals we did not consider high numbers of repeats here (although obviously we expect this pattern of diminishing gains to continue).

Below we have appended R code to execute the power analysis described above for a specified number of individuals (n), observations per individual (o), true value of repeatability (sig2I), and number of simulated data sets (i). This code is dependent on ASReml-R (a proprietary package) but the second section could be readily modified to use alternative (freely available) packages for fitting the linear mixed effect model available from CRAN.

#1)SPECIFY PARAMETERS AND SIMULATE PHENOTYPIC OBSERVATIONS

#unit variance so among individual variance (sig2I) = repeatability

#residual variance (sig2R) is therefore = 1-sig2I

sig2I <- 0.35 # repeatability

n <- 30 # number of individuals

o <- 4 # number of observations per individual

i <- 200 # number simulated data sets

sig2R <-(1- sig2I)

results<-matrix(0, i, 8)

for (i in 1:i) {

ID<-as.vector(rep(1:n, o))

ind<-rnorm(n, mean=0, sd=(sig2I^0.5))

ind2<-rep(ind,o)

res<-rnorm((n*o), mean=0, sd=(sig2R^0.5))

Y<-ind2+res #vector of simulated trait observations

simdat<- as.data.frame(cbind(ID, ind, res, Y))

colnames(simdat)<-c("ID", "ind" ,"res", "Y")

simdat$ID<-as.factor(simdat$ID)

#2) FITS AND COMPARES MODELS FOR EACH SIMULATED DATA SET

library(asreml)

model1<-asreml(fixed=Y~1, random=~ID, data=simdat, maxiter=20)

model2<-asreml(fixed=Y~1, data=simdat, maxiter=20)

VI<-summary(model1)$varcomp$component[1] # extract VI

VR<-summary(model1)$varcomp$component[2] # extract VR

Rep<-VI/VI+VR) #estimates repeatability

LogL1<-model1$loglik

LogL2<-model2$loglik

chi2<- 2*(model1$loglik-model2$loglik)

P1<- 0.5*(1-pchisq(2*(model1$loglik-model2$loglik),1))

P2<- 2*P1

results[i,]<-cbind(VI,VR,Rep,LogL1,LogL2,chi2,P2,P1)

print(i)

}

results<-as.data.frame(results)

colnames(results)<-c("VI","VR","Rep","LogL1","LogL2","chi2","P2","P1")

#3) OUTPUT RESULT SUMMARY AND ESTIMATED POWER (1 AND 2 TAILED TESTS)

hist(results$R) #gives histogram of repeatability estimates

mean(results$R) #gives mean of repeatability estimates

POWER1tail<-length(results$P1[results$P1<=0.05])/length(results$P1)

POWER2tail<-length(results$P2[results$P2<=0.05])/length(results$P2)

POWER1tail #returns power estimate for 1-tailed test, alpha = 0.05

POWER2tail #returns power estimate for 2-tailed test, alpha = 0.05
