# Supplementary material for: Integrating Personality Research and Animal Contest Theory: Aggressiveness in the Green Swordtail Xiphophorus helleri
Source: PLoS One. 2011 Nov 30;6(11):e28024. doi: 10.1371/journal.pone.0028024 (PMC3227624; doi:10.1371/journal.pone.0028024)
Supplement: Appendix S2 — Ethogram of behaviours assayed in mirror tests and dyadic interaction tests. (DOC) [file pone.0028024.s003.doc]

**Supplemental Appendix 2: Ethogram of behaviours assayed in mirror tests and dyadic interaction tests**

| **Behaviour** | **Description** |
| --- | --- |
| **Mirror tests** | |
| *Approach* | Focal swims in a forward motion toward mirror at a constant speed and stops when in proximity (max. 5 cm). |
| *Display* | Focal displays lateral surface of the body to mirror from a maximum distance of 10cm. Focal may contort into a sigmoid (S) or C-shaped curve (with concave surface facing the mirror) and may also tilt their lateral body surface. |
| *Attack* | Sudden forward acceleration of the Focal towards the mirror which may or may not result in brief contact by the mouthparts |
| **Dyadic interaction tests** | |
| *Approach* | Focal swims in a forward motion toward opponent at a constant speed, starting from a maximum of half a tank length and stopping in proximity to opponent. |
| *Tail beat* | Single distinct sideways beat of the Focal caudal fin. Maximum separation of Focal and Opponent of 10cm. |
| *Display* | Focal displays lateral surface of the body to opponent from a maximum distance of 10cm. Focal may contort into a sigmoid or C-shaped curve (with concave surface facing the opponent) and may also tilt their lateral body surface. Display may or may not be mutual, with the two fish oriented laterally (either parallel or anti-parallel). |
| *Attack* | Sudden forward acceleration of the Focal towards the opponent, starting from a maximum of half a tank length away, that may or may not result in contact and/or bite hold. |
| *Retreat* | Focal swims away from Opponent at constant speed without acceleration from an initial distance separation of maximum 5cm. Swimming may be either forwards or backwards. |
| *Flee* | Focal swims suddenly and rapidly away from Opponent from an initial maximum separation of 10cm and covering a minimum distance of half a tank length. |
